# Effects of steroid stewardship on glycemic control in acute exacerbations of chronic obstructive pulmonary disease patients

**DOI:** 10.1111/crj.13613

**Published:** 2023-04-13

**Authors:** Jennifer L. Cole

**Affiliations:** ^1^ Veterans Healthcare System of the Ozarks Fayetteville Arkansas USA

**Keywords:** AECOPD, corticosteroids, hyperglycemia, stewardship

## Abstract

**Introduction:**

The adverse effects of corticosteroids are dose‐dependent, and guidance is to use the lowest effective dose in most disease states. The study facility recently reported a steroid stewardship program that reduced steroid dosing in acute exacerbations of chronic obstructive pulmonary disease (AECOPD) patients by 50%. The purpose of this post‐hoc analysis was to evaluate the effect of this intervention on glycemic control in hospitalized AECOPD before and after cohorts.

**Methods:**

This was a retrospective post‐hoc review of hospitalized patients in a before and after study design (*n* = 27 in each group). The primary endpoint was the proportion of glucose readings >180 mg/dL. Baseline characteristics, mean glucose levels, and corrective insulin were also collected. Continuous variables were compared with a Student's t‐test (or Mann–Whitney U where appropriate) and nominal variables with a chi‐square test in R Studio.

**Results:**

There was a significantly higher proportion of glucose >180 mg/dL readings in the pre‐intervention cohort: 38% vs. 25% (*p* = 0.007). The mean glucose levels were numerically lower post‐intervention but did not reach statistical significance (160 mg/dL vs. 145 mg/dL, *p* = 0.27) both in diabetics (192 mg/dL vs. 181 mg/dl, *p* = 0.69) and non‐diabetics (142 mg/dL vs. 125 mg/dL, *p* = 0.08). The use of correctional insulin was similar: a median of 25 units vs. 24.5 units (*p* = 0.92).

**Conclusion:**

A stewardship program focused on steroid reduction in AECOPD significantly lowered the proportion of hyperglycemic readings but did not significantly affect mean glucose and corrective insulin usage while hospitalized.

## Graphical abstract

A post‐hoc analysis of a steroid stewardship program evaluated the effect of this intervention on glycemic control in AECOPD patients. Steroid stewardship decreased the proportion of glucose readings >180 mg/dL in hospitalized patients. Mean glucose readings were lower but did not reach statistical significance.

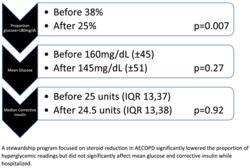

## 1 INTRODUCTION

Corticosteroids are the most common cause of drug‐induced hyperglycemia, with a reported incidence of 64% to 86% of hospitalized patients, with 70% achieving glucose levels greater than 180 mg/dL.^1^, ^2^ The severity of hyperglycemia can be profound, with reported glucose levels up 68% compared with baseline, and diabetic patients are particularly affected.^3^ The clinical impact of steroid‐induced hyperglycemia during hospitalization has been studied, with affected patients having higher odds of a combined endpoint of mortality, cardiovascular events and infections compared with normoglycemic patients.^4^

A joint consensus report from the American Association of Clinical Endocrinologists, the American Association of Diabetes Educators, the American Diabetes Association (ADA), the Endocrine Society, and the Pediatric Endocrine Society defined a glucose level greater than 180 mg/dL as ‘clinically meaningful’ hyperglycemia.^5^ Therefore ADA standards for the care of hospitalized patients recommend this level as a threshold for initiating insulin therapy. Managing inpatient hyperglycemia is heavy on facility resources, with an estimated $97 Billion spent on inpatient diabetic services annually.^6^

The adverse effects of steroids are dose‐dependent, and guidance is to use the lowest effective dose in most disease states.^3^ Moreover, the CHEST Foundation recently advocated for steroid stewardship, given the unwanted effects of cumulative exposure.^7^ In acute exacerbations of COPD (AECOPD), consensus guidelines recommend low‐dose oral steroids equivalent to prednisone 40 mg.^8^ Despite this guidance, higher than recommended steroid dosing is commonly reported in real‐world settings.^9^, ^10^, ^11^

Researchers at the study facility previously improved guideline adherence with a successful pharmacist‐led steroid stewardship intervention. Prescribed prednisone equivalents in hospitalized AECOPD patients were reduced by approximately 50% through a multifaceted intervention consisting of (1) engaging providers with an anonymous survey and clinical case, (2) face‐to‐face education with peer‐comparison and (3) prospective audit and feedback from a clinical pharmacist (results previously reported).^12^, ^13^ The reduction in steroid dosing was not associated with any known deleterious effects, but questions remain about clinical benefits. The objective of this post‐hoc analysis was to determine the effects of a steroid stewardship program on glycemic outcomes in hospitalized AECOPD patients.

## 2 METHODS

The study facility is a 52‐bed primary Veterans Affairs hospital. The Licensed bed total includes seven ICU beds and 28 medical/surgical acute care beds. There is one clinical pharmacist that rounds in the inpatient wards Monday through Friday, 0730 through 1600.

The original steroid stewardship evaluation was a quasi‐experimental before and after study conducted with a retrospective chart review.^12^ Patients were randomly selected from two 12‐month treatment periods: before group April 2019–March 2020 and after group May 2020–April 2021. The original study was powered (80%) to detect a 50% reduction in mean daily prednisone equivalents. Targeted patients for inclusion were adults hospitalized for AECOPD for at least 24 h and receiving systemic steroids. All patients in the original study were included in this post‐hoc analysis with no exclusions (*n* = 27 in each cohort). The primary outcome is the proportion of glucose readings greater than 180 mg/dL during the index admission. Secondary outcomes include mean glucose levels while hospitalized and the use of corrective insulin. Outcomes are reported in all‐comers, then further divided into patients that were prescribed corrective insulin as needed and those who were not prescribed corrective insulin.

### 2.1 Data collection

The majority of baseline characteristics and indicators of disease severity were collected in the original study.^12^ Additional data collected included hemoglobin A1c at the time of the index admission, all glucose readings during the index admission, including both serum laboratory draws and point‐of‐care (fingerstick) capillary tests, body mass index and corrective insulin doses. Basal and mealtime insulins were recorded, including adjustments during hospitalization (dose adjustments, hold orders and patient refusals). Non‐insulin diabetic medications used during hospitalizzation were also recorded: metformin, sulfonylureas, dipeptidyl peptidase‐4 inhibitors, thiazolidinediones, sodium‐glucose cotransporter‐2 inhibitors and glucagon‐like peptide‐1 receptor antagonists. Any use of TPN, PPN, insulin pumps or insulin infusions was noted. The incidence of hypoglycemia (defined as blood glucose less than 70 mg/dL) and the use of rescue medications were also recorded (D10W, D50W and glucagon). Barcoded medication reports were used for all medication data collection.

Standardised algorithms at the study facility for corrective insulin are listed in Table 1. Corrective scales for the general wards differ from ICU scales. Facility guidance for ICU patients is to advance to the next higher regimen if all readings are greater than 100 mg/dL within 24 h and there are two consecutive readings greater than 160 mg/dL. Guidance is to decrease to the next lower regimen if glucose readings are less than 80 mg/dL two times within 12 h. All algorithms utilize regular human insulin. Capillary glucose checks default to four times a day with meals (0700, 1200, 1800, 2200). Facility guidance for an insulin infusion (non‐DKA) is to initiate in ICU patients if two consecutive blood glucose readings are greater than 180 mg/dL and not controlled by high‐dose corrective doses.

**TABLE 1:** Standardised corrective insulin algorithms. All glucose readings in mg/dL. All units are for regular human insulin. Capillary glucose checks default to four times a day with meals (0700, 1200, 1800, 2200).

Standard sliding scale general ward	Low dose ICU only	Medium dose ICU only	High dose ICU only	Very high dose ICU only
150–199 mg/dL = 2 units	126–150 = 2 units	126–150 = 2 units	110–125 = 2 units	110–125 = 2 units
200–250 mg/dL = 4 units	151–175 = 3 units	151–175 = 4 units	126–150 = 4 units	126–150 = 5 units
251–300 mg/dL = 6 units	176–200 = 4 units	175–200 = 5 units	151–175 = 6 units	151–175 = 7 units
301–350 mg/dL= 8 units	201–225 = 5 units	201–225 = 7 units	176–200 = 8 units	176–200 = 10 units
351–400 mg/dL = 10 units	226–250 = 6 units	226–250 = 8 units	201–225 = 10 units	201–225 = 12 units
401–450 mg/dL = 12 units	251–275 = 7 units	251–275 = 10 units	226–250 = 12 units	226–250 = 15 units
>450 mg/dL = call MD	276–300 = 8 units	276–300 = 11 units	251–275 = 14 units	251–275 = 17 units
	301–325 = 9 units	301–325 = 13 units	276–300 = 16 units	276–300 = 20 units
	>325 = 10 units and call MD	>325 = 14 units and call MD	301–325 = 18 units >325 = 0 units and call MD	301–325 = 22 units >325 = 25 units and call MD

### 2.2 Statistical analysis

Normally distributed outcomes are represented as mean ± standard deviation (SD), whereas non‐normally distributed outcomes are represented as the median and interquartile range (IQR). Continuous outcomes were analyzed with a Student's t test or Mann–Whitney U where appropriate. Nominal outcomes were analyzed with a chi‐square or Fisher's exact where appropriate. All comparisons were analyzed in R Foundation for Statistical Computing version 3.6.3 (Vienna Austria). These methods were reviewed by the facility's Institutional Review Board and granted non‐research status, waiving the requirement for informed consent (IRB00006264).

## 3 RESULTS

The original cohorts were well matched in terms of COPD severity at baseline, the severity of exacerbation and comorbid conditions.^12^ Pertinent baseline findings are reported in Table 2. In glycemic‐related outcomes, groups were well matched in terms of baseline hemoglobin A1c and body mass index (Table 3).

**TABLE 2:** Baseline characteristics from original study.^12^

Variable	Before *n* = 27	After *n* = 27	*P* value
Male (%)	27 (100)	26 (97)	
Diabetes mellitus (%)	10 (37)	14 (52)	0.27
ICU admission (%)	8 (30)	9 (33)	0.77
GOLD classification n (%)			
Class I	2 (7)	0	‐
Class II	8 (30)	8 (30)	1
Class III	8 (30)	8 (30)	1
Class IV	4 (15)	5 (18)	0.75
FEV‐1 not recorded	5 (18)	6 (22)	0.74
PaO_2_/FiO_2_ median (IQR)	217 (194, 257)	254 (190, 283)	0.52
Arterial pH ≤ 7.33, *n* (%)	3 (11)	4 (15)	0.69
Non‐invasive ventilation *n* (%)	2 (7)	5 (18)	0.22
Mechanical ventilation	0	0	‐
APACHE II score, median	14 (13, 16)	10 (5, 17)	0.20
Charlson Comorbidity index, median	5 (4, 6)	7 (4, 8)	0.20

Abbreviations: APACHE, acute physiology and chronic health evaluation; GOLD, global initiative for chronic obstructive lung disease; FEV‐1; forced expiratory volume in 1 s.

**TABLE 3:** Outcomes.

Outcome (all patients)	Before *n* = 27	After *n* = 27	*P* value
Mean daily prednisone equivalents	118 mg (±88)	53 mg (±26)	0.0003
Duration (days) median	8 (6, 12.5)	7 (5, 8)	0.44
Age (years), mean	71	70	0.44
Body Mass Index, mean	28.6 (±6.1)	28.4 (±8.2)	0.91
Length of stay (days), mean	3.3 (±1.4)	3.9 (±2.1)	0.21
Mean A1c %	6.6 (±1.4)	6.4 (±1.2)	0.47
Mean glucose (mg/dL)	160 (±45)	145 (±51)	0.27
Median number of glucose checks	4 (3, 7.5)	4 (2, 9.5)	0.48
Proportion >180 mg/dL	38%	25%	0.007

Prescribed correctional insulin	Before *n* = 10	After *n* = 10	
Mean daily prednisone equivalents	124 mg (±107)	50 mg (±23)	0.04
Age (years)	70	72	0.34
Body mass index, mean	30.3 (±5.3)	31.7 (±6.9)	0.62
Length of stay (days), mean	3.2 (±1.3)	4.4 (±1.8)	0.11
Mean A1c %	7.7 (±1.6)	7.2 (±1.7)	0.50
Mean glucose (mg/dL)	192 (±53)	181 (±65)	0.69
Median number of glucose checks	10 (7.25, 12.75)	11 (9.25, 22)	*p* = 0.88
Proportion >180 mg/dl	49%	31%	0.004
Median number of SSI doses	6 (2, 8)	5 (4, 9)	0.88
Median SSI correctional dose, units	25 (13, 37)	24.5 (13, 38)	0.92
Basal insulin, *n*	5	4	0.65
Basal adjustments (total)	6	14	‐
Mealtime insulin, *n*	0	1	‐
Metformin, *n*	3	4	‐
Thiazolidinedione, *n*	1	0	‐
Sulfonylurea, *n*	5	0	‐

No correctional insulin	Before *n* = 17	After *n* = 17	
Mean daily prednisone equivalents	119 mg (±78)	55 mg (±28)	0.004
Age (years), mean	70	70	0.96
Body mass index	27.6 (±6.5)	26.4 (±8.5)	0.66
Length of stay (days)	3.4 (±1.4)	3.6 (±2.3)	0.66
Mean A1c %	5.9 (±1.5)	5.8 (±0.41)	0.54
Mean glucose (mg/dL)	142 (±28)	125 (±25)	0.08
Median number of glucose checks	3 (1, 4)	3 (1, 4)	0.57
Proportion >180 mg/dL	17%	9%	0.17

Abbreviation: SSI, sliding scale insulin.

The overall proportion of glucose readings greater than 180 mg/dL was significantly lower in the post‐intervention group (38% vs. 25%, *p* = 0.007). This was predominantly driven by diabetic patients prescribed corrective insulin (49% vs. 31%, *p* = 0.004). Average glucose readings per hospitalization were lower in the post‐intervention group (160 mg/dL (±45) vs. 145 mg/dL (±51), *p* = 0.27), but this did not reach statistical significance in any category. There was a larger difference in means noted in non‐diabetic patients not prescribed correctional insulin (142 mg/dL (±28) vs. 125 mg/dL (±25), *p* = 0.08). The median number of correctional insulin doses given was similar (6 doses (IQR 2.8) vs. 5 doses (IQR 4.9), *p* = 0.88). Additionally, the median cumulative correctional insulin dosing was not significantly different between groups (25 units (IQR 13.37) vs. 24.5 units (IQR 13.38), *p* = 0.92).

There was one hypoglycemic event in the before group and two in the after group. There was one incident of treatment with D50W in the after group (none in the before group). There were no cases of TPN, PPN, insulin infusion or insulin pumps in either cohort.

## 4 DISCUSSION

This study is important as it is the first to demonstrate clinical benefits with intentional stewardship directed at steroid dosing. There were no specific interventions targeting glucose management outside of routine clinical practice during either cohort. Therefore, any clinical improvements would ostensibly be contributed to improved guideline adherence.

The glycemic endpoints studied were the most pragmatic in routine clinical practice. Other clinical effects of reducing steroid doses were beyond the scope of this study, such as fluid retention, modulated immune response, dyslipidemia, changes in sleep patterns and mood disorders. Additionally, these endpoints are difficult to define and pool in meta‐analyses; therefore, targeted prospective studies may be needed to demonstrate such benefits.

In addition to pneumonia, stroke and ischemic heart disease, AECOPD has demonstrated better outcomes with lower serum glucose readings. Baker et al. reported a higher relative risk of death and longer length of hospital stay in patients hospitalized for AECOPD who had higher serum glucose readings compared with normoglycemic patients. This was independent of age, sex, previous diabetes diagnosis and COPD severity. Further, for each 1 mmol/L (18 mg/dL) increase in serum glucose, adverse outcomes increased by 15% (95% CI 4–27, *p* = 0.006).^14^

The trend toward lower mean glucose readings in the non‐diabetic cohort is an interesting finding, as this may have an impact on the phenomenon of converting these patients to new diabetics.^15^, ^16^ The odds ratio for converting non‐diabetics to new diabetics is reported between 1.5–2.5 with glucocorticoid use, with higher doses and duration being strong predictors of diabetes induction.^17^ Of note, the proportion of hyperglycemic findings was nearly cut in half (17% vs. 9%). While this study did not reach statistical significance, larger cohorts may be needed to adequately reflect this finding.

The benefit in hyperglycemic proportions seen in the diabetic cohort is promising as these patients may be especially susceptible to corticosteroid side effects.^3^ The lack of improvement seen in correctional insulin usage could be attributed to the low number of patients captured by the study methods. Additionally, changes in individual provider practices could have contributed here (e.g., reluctance to change to a higher dose insulin sliding scale or more aggressive changes to basal insulin dosing).

The study findings may be of particular importance in quality benchmarks for healthcare facilities.^18^ Within the VA, the Inpatient Evaluation Center (IPEC) measures risk‐adjusted outcomes for hospitalized patients for quality and safety, including hyperglycemic events. The Centers for Medicare and Medicaid Services recently added new inpatient glycemic management metrics around hypoglycemia and hyperglycemia.^19^ Additionally, time in range‐centered care (serum glucose between 70 and 180 mg/dL) is gaining recognition as a therapeutic benchmark in diabetic patients.^5^, ^20^

The average glucose levels reported are similar to other published reports in diabetics and non‐diabetics with AECOPD.^21^ While the glycemic improvements reported herein may seem pedestrian, it is important to recognize that the original study was a proof‐of‐concept project powered for changes in steroid dosing.^12^ Although these patients were not randomly selected for the post‐hoc analysis, it should be noted that the primary outcome found would require *n* = 398 patients to achieve the power of 80%; therefore, a larger enrollment capacity would be needed. In observational ‘big data’ analytics, these findings may be more profound. In an epidemiologic study comparing high‐dose and low‐dose steroid regimens in AECOPD, hyperglycemia was more common in the high‐dose group. Moreover, after propensity matching, the high‐dose steroid group was significantly more likely to receive insulin therapy during their admission.^22^ Finally, even small changes in glucose control may prove to be beneficial.^14^

### 4.1 Limitations

There are several limitations to consider when interpreting these results. The quasi‐experimental study design does not control for potential confounders affecting the outcomes. While there is facility guidance for the treatment of hyperglycemia, this is not strictly enforced, and providers may have variable treatment practices. Most diabetics at the study facility are prescribed a low to moderate carbohydrate diet; however, this data was not collected, and there may be variations in intake overall. Variations in patient inflammatory responses and underlying insulin resistance can also affect serum glucose levels. It should be noted, however, that patients in the original study were randomly selected to help account for some of these confounders.

The relatively small sample size brings up concerns for type I and type II errors in the post‐hoc analysis. The original study was powered for a reduction in steroid dosing, not for changes in clinical outcomes. External validity should also be considered, as the steroid doses in the pre‐intervention cohort were quite high and still averaged above guideline‐recommended doses in the post‐intervention cohort.

## 5 CONCLUSION

Promoting guideline adherence with a steroid stewardship project significantly improved the incidence of hyperglycemia in hospitalized AECOPD patients. The effect seen in diabetic patients was the major driver for this outcome. The mean daily glucose readings and corrective insulin dosing were not significantly affected. Larger cohorts will be needed to more clearly demonstrate these secondary glycemic endpoints along with other clinical benefits of lower steroid doses. Nonetheless, the project methodology is easily reproducible and readily implementable for facilities with larger enrollment capacities.

## AUTHOR CONTRIBUTIONS

Jennifer L. Cole was responsible for the study concept, design and data collection. She was the sole author of the manuscript.

## CONFLICT OF INTEREST STATEMENT

The author declares that there is no conflict of interest. The views and opinions expressed in this article are those of the author and do not reflect the views of the Department of Veterans Affairs or any other US government agency.

## ETHICS STATEMENT

The methodology used resulted in minimal harm and did not meet the definition of human subjects research as defined in VHA directive 1200.05 protecting human subjects. Data handling was in compliance with VHA Office of Research and Development directive 1200.12.

## Data Availability

The data that support the findings of this study are openly available upon request.
